# 2-Hour Postload Serum Glucose Levels and Maternal Blood Pressure as Independent Predictors of Birth Weight in “Appropriate for Gestational Age” Neonates in Healthy Nondiabetic Pregnancies

**DOI:** 10.1155/2013/757459

**Published:** 2013-09-18

**Authors:** Jumana Saleh, Lovina Machado, Zahra Razvi

**Affiliations:** ^1^Biochemistry Department, College of Medicine, Sultan Qaboos University, P.O. Box 35, 123, Muscat, Oman; ^2^Obstetrics and Gynecology, College of Medicine, Sultan Qaboos University Hospital, P.O. Box 35, 123, Muscat, Oman; ^3^College of Medicine, Sultan Qaboos University, P.O. Box 35, 123, Muscat, Oman

## Abstract

*Introduction*. Increased neonatal birth weight (NBW), often associated with diabetic pregnancies, is a recognized indicator of childhood obesity and future metabolic risk. Predictors of NBW in healthy non-diabetic pregnancies are not yet established. Here, we investigated the association of maternal parameters of healthy non-diabetic mothers with NBW of their “appropriate-for-gestational age” neonates. *Methods*. The study involved 36 healthy mother/infant pairs. Examined parameters included NBW, maternal age, first and last trimester (BMI), weight gain, fasting serum lipids and glucose, 2-hour postload glucose levels and blood pressure. *Results*. Postload-glucose levels were significantly higher in mothers of heavier neonates. ANOVA results indicated that 15% increase in postload-glucose levels corresponded to more than 0.5 Kg increase in NBW in the third tertile. NBW correlated positively with postload glucose levels, and negatively with systolic blood pressure. Regression analysis showed that the main predictors of NBW were postload-glucose levels (*B* = 0.455, *P* = 0.003), followed by systolic blood pressure (*B* = −0.447, *P* = 0.004), together predicting 31.7% NBW variation. *Conclusion*. This study highlights that increased maternal postload sugar levels and blood pressure, within the normal range, highly predicts NBW of healthy mothers. These findings may provide focus for early dietary intervention measures to avoid future risks to the mother and baby.

## 1. Introduction 

During the past few decades, the number of obese children has been rising worldwide [1]. Childhood obesity is a major threat to the child's future health as it is linked to several complications including cardiovascular disease [2], sleep apnea, type 2 diabetes, asthma, cancer, and psychosocial disorders [3, 4]. A major indicator of childhood obesity is birth weight [5]. Several maternal factors have been shown to positively correlate with increased neonatal birth weight (NBW) including maternal age, weight gain during pregnancy [6], genetic predisposition, maternal glucose, and lipid levels [7, 8]. On the other hand, smoking and hypertension showed negative associations [9]. Maternal hypertension has been commonly linked to the birth of small for gestational age (SGA) neonates, which was reportedly linked to hypertension and metabolic complications during adulthood [9, 10]. It is well established that gestational diabetes causes increased fetal adiposity and high birth weight; therefore, most studies focus on large for gestational age (LGA) infants as an outcome of diabetic pregnancies [11]. However, increased birth weight is not restricted to diabetic pregnancies, as nondiabetic healthy mothers also deliver large babies, and maternal glucose variations, even within the normal range, may predict future metabolic risk [12–14]. A continuous debate remains regarding maternal factors that predict birth weight in healthy nondiabetic pregnancies with normal glucose tolerance [8, 15, 16]. A study by Ludwig and Currie showed that birth weight was increased with pregnancy weight gain independent of genetic factors [4]. This was attributed to increased insulin resistance as the pregnancy progresses and was supported by several studies [6, 17]. More reports showed positive associations with other maternal weight parameters including prepregnancy BMI [6, 18, 19], first trimester BMI [20], and maternal BMI from midpregnancy onwards [6]. Triglyceride levels rise markedly during mid-late pregnancy in the form of accumulating TG-rich carrying lipoproteins, mainly very low density lipoproteins (VLDLs) [21]. Maternal TG levels were shown to positively correlate with birth weight in several studies [22–24]. Studies by Knopp et al. and others found positive correlations between maternal TG levels and birth weight that were independent of glucose levels or maternal weight [25, 26]. The mechanism by which maternal hyperlipidemia affects fetal growth is not yet clear. Maternal serum TGs do not cross the placenta, and the transfer of hydrolyzed fatty acids is relatively limited compared to glucose diffusion across the placenta [21]. However, maternal triglyceride hydrolysis is enhanced by placental lipoprotein lipase, which is active during late gestation, thereby increasing the fatty acid flux to the fetal circulation. Fatty acid transport into the fetal milieu occurs through the placental villous trophoblast by rapid simple diffusion. Moreover, fatty acid binding proteins (FABPs) have been identified that they may facilitate fatty acid transfer across placental membranes to the fetal circulation [21]. Consequently, fetal fat storage is enhanced by fatty acid trapping and activation of TG synthesis pathways mediated by fetal insulin and endogenous factors [23].

On the other hand, maternal glycemic status received the most attention in the literature. Glycemic parameters that showed positive associations with birth weight include serum fasting glucose, postprandial glucose, and postload glucose levels [27–32]. Generally, large scale studies constitute a broad range of inclusion criteria and use different experimental settings, mostly based on retrospective analysis. This may explain inconsistent observations and various findings of maternal predictors. Moreover, most studies investigating associations between maternal parameters and birth weight justifiably focus on parameters of apparent clinical significance, particularly, abnormal maternal and neonatal anthropometric or metabolic profiles. In this prospective study, we aim to investigate predictors of normal birth weight variations in a controlled setting of carefully selected healthy nonhypertensive, nondiabetic women screened for normal glucose tolerance and normal pregnancy weight gain. All women were from similar cultural backgrounds, nonsmokers, and did not consume alcohol. 

## 2. Study Design and Data Collection 

This is a cross-sectional study including 36 mother-infant pairs. The study target was pregnant Omani healthy, nondiabetic mothers during their third trimester coming for follow-up visits at the Obstetrics and Gynecology Department at Sultan Qaboos University Hospital (SQUH). Mothers scheduled for appointments were called by phone to obtain an initial verbal consent after explaining the aims and methodology of the project. Before their appointment, the women followed a controlled diet containing at least 200 grams of carbohydrates per day for 3 days. On the day of the appointment, the women came fasting overnight for the lipid profile and oral glucose tolerance test (OGTT) (fasting and 2 hr postload glucose of 75 grams). The research methodology was clearly explained to the participating women, and an informed consent was obtained. Anthropometric data including age, height, number of pregnancies, and medical history were taken from hospital electronic records and pregnancy cards. The initial weights of all mothers were recorded during the first antenatal visit at 8–10 weeks of gestation. Maternal weight and height were used to calculate maternal body mass index (BMI) (Kg/m^2^). The study subjects were weighed with minimum clothing using the same mechanical column scale at each visit. Serum fasting glucose levels followed by OGTT were performed during the appointment scheduled for blood tests at 26–30 weeks of gestation. Mothers with high OGTT levels (>5.5 mmol/L fasting and >7.8 mmol/L 2 hr postload glucose) were excluded as impaired glucose tolerance or gestational diabetes according to guidelines followed by SQUH [33]. A fasting blood sample for lipid profile was also obtained during the same visit. The last maternal weight and blood pressure before delivery at 34–36 weeks were later obtained from the hospital records. After delivery, the birth weights of the babies were taken from the maternity register. Unclothed newborns were weighed immediately after delivery using an electronic weighing scale. 

### 2.1. Selection Criteria

 The pregnant women selected were all Omani, nondiabetic, nonhypertensive, and not suffering from any metabolic disorder. None of the women were smokers or consumed alcohol. Only mothers with singleton pregnancies were included in the study. The research was approved by the Medical Research Ethics Committee at Sultan Qaboos University. Newborns selected were delivered vaginally and were all healthy and appropriate for gestational age (AGA). Their birth weights were within the normal range (2.5–4 Kg) [8, 34].

### 2.2. Biochemical Analysis

The biochemical analysis was performed in the clinical biochemistry laboratory at SQUH. The lipid profile parameters measured included total cholesterol, high density lipoproteins, low density lipoproteins, and triglycerides. Analysis was performed using an automated clinical chemistry analyzer (Cobas Integra 800, Roche Deutschland) based on enzymatic colorimetric assays for lipid analytes including triglycerides, total cholesterol, HDL, and LDL. Precision of biochemical measurements using the Cobas analyzer was determined by using human control samples (within-run *n* = 20, and between-run *n* = 20). Within-run precision coefficients of variations (CVs) were below 2%, and between-run CVs were below 2.5% regarding all biochemical analytes included in the study which indicated satisfactory accuracy and precision.

### 2.3. Data Analysis

 All the data was analyzed using the statistical program SPSS (version 19). One sample Kolmogorov-Smirnov (*K-S* test) was used to determine the parameters that were normally distributed. Results were expressed as mean ± SD or median unless specified. Two-tailed significance was set to *P* < 0.05. To compare specific parameters between male and female newborns, independent sample *t*-test was used. Bivariate correlation analysis was applied to determine significant associations between the maternal parameters and birth weight of the newborn, and significance was set as *P* < 0.05. Neonatal birth weights (NBW) were categorized in tertiles. Differences in maternal parameters across tertiles were evaluated by one way analysis of variance (ANOVA). Tukey's test was used for post hoc comparison of means between each pair of groups. Stepwise multiple regression analysis was performed to determine maternal predictors of NBW. 

## 3. Results

The mean values of anthropometric and biochemical parameters measured in the study for the mothers and infants are presented in Table 1. None of the women were initially underweight. The weight gain for all women was in the acceptable range considering their BMI status [7, 35]. The age of the women ranged between 21 and 41 years. No significant difference was found in NBW between male and female newborns. The neonate's birth weight range (2.7–3.92 Kg) was divided into tertiles (Table 2). One way ANOVA analysis showed an overall significant difference in maternal postload glucose levels across NBW tertiles (*P* = 0.029). The major increase was found in maternal postload glucose levels of mothers of babies in the third tertile (Table 2). These findings indicate that 15% increase in postload glucose levels corresponds to 0.54 Kg increase in NBW in the third tertile compared to NBW means in the first and second tertiles. On the other hand, although maternal TG levels, initial BMI, and weight gain showed higher trends for birth weights in the third tertile, differences did not reach statistical significance (Table 2). Bivariate correlations showed that the only significant association was the positive correlation between maternal postload glucose levels and NBW (Table 3). Multiple regression analysis was performed to determine predictors of increased NBW. Neonatal birth weight was set as the dependent parameter, and all measured maternal anthropometric and biochemical parameters were set as independent variables. Multiple regression analysis indicated that postload glucose levels were the major predictor of NBW (*B* = 0.455, *P* = 0.003) followed by systolic blood pressure (*B* = −0.447, *P* = 0.004), together predicting 31.7% variation in NBW as indicated by  *R* ^2^. Other maternal factors were excluded from the regression model as nonsignificant predictors (Table 4).

## 4. Discussion 

The major findings in this study were that the maternal 2 hr postload glucose levels, and systolic blood pressure within the normal range, positively correlated, and independently predicted, NBW variations in nondiabetic pregnancies. Analysis of variance (ANOVA) showed that only 15% increase in maternal postload glucose levels corresponded to more than 0.5 Kg increase in NBW in the third tertile compared to lower birth weight tertiles. In spite of increasing trends in maternal BMI, weight gain, age, and fasting glucose corresponding to birth weight in the third tertile, the differences were not significant. The positive association between 2 hr postload glucose and the risk of delivering LGA neonates was a common observation in several studies without adjustment for covariates [36–39]. A few studies reported a strong association after covariate adjustments [28–32]. Weak or nonsignificant relationships between postload glucose and the risk of LGA were also reported [29]. Other studies reported positive correlations between birth weight and fasting glucose levels [12, 13, 40, 41]. As the 75 g OGTT can be regarded as a surrogate marker of meal postprandial glycemia [42], our findings agree with previous findings in that mild elevations in glucose tolerance, or mild dietary glucose elevations within the normal range, may result in excessive fetal growth [14]. Our findings may be explained by the Pedersen hypothesis, which suggests that maternal hyperglycemia (even within the normal range) increases fetal insulin levels, leading to accelerated fetal growth [43]. As insulin resistance normally develops during pregnancy, increased flux of dietary maternal glucose to the fetal circulation stimulates fetal insulin release. As a potent fat storage hormone, insulin enhances uptake of glucose and lipids by fetal adipose tissue causing increased weight gain [44]. The effect of postload or postprandial glucose may be more pronounced than lipids due to the rapid transfer of maternal glucose through the placental membrane to the fetus compared to fatty acids that may require specialized placental transport mechanisms [45]. Eventually, both energy forms largely affect fetal weight. This may not be apparent with fasting glucose levels as usually maternal fasting glucose does not increase significantly during late gestation. On the contrary, fasting glucose levels tend to decrease possibly due to increased fetal demands [23]. Furthermore, studies that have shown that maternal weight gain and TG levels are significant predictors of NBW have not included postprandial or postload glucose as independent variables [4, 6, 17, 23]. Also blood samples collected in some studies were nonfasting for lipid analysis or were obtained at different time points during the pregnancy period [22, 46]. Interestingly, in support of our findings a recent large study in Norway covering the time period 1989–2010 showed that birth weight trends changed in parallel to consumption of sucrose largely consumed as sugar-sweetened beverages, while the known predictors of birth weight could not explain the observed temporal changes in birth weight. The study suggested that increased fetal growth may be partly explained by a direct effect of rapidly absorbable sugar, largely from high and frequent intakes of sugar-sweetened beverages independent of effects of maternal BMI and gestational weight gain [47]. 

The main strengths of this study were restricting the inclusion criteria to nondiabetic women with normal glucose tolerance, blood pressure, and maternal weight gain. Alcohol consumption and smoking were nonexistent among the study subjects. Importantly also, this study was a prospective study as fasting triglyceride levels are not routinely measured at late gestation for nondiabetic women, an advantage not available in retrospective studies where lipid measures were not included as independent variables. In addition, variability in neonatal birth weight in this study was limited to AGA neonates in an attempt to avoid unforeseen adverse neonatal outcomes of small for gestational age or large for gestational age neonates and may introduce unexpected variables to the study. Furthermore, homogeneity was relatively controlled considering lifestyle and cultural backgrounds by restricting the study to Omani women. Limitations of this study were unavoidable as some women delivered elsewhere, and subjects with missing data were excluded. It was also not possible to obtain a reliable prepregnancy weight measures from women as they usually attend the clinic only after confirming their pregnancy. However, the weight gain during the first trimester is usually minimal [48], and this measure was adopted by previous studies [20, 49].

Our result showing systolic blood pressure as a predictor of decreased NBW is very well in agreement with previous observations of an inverse association between fetal growth and maternal blood pressure. Maternal blood pressure has been linked to fetal growth retardation [9, 10]. The biochemical nature of this association remains unclear. However, it has been suggested that exposure to this adverse environment during fetal development is associated with increased cardiometabolic risk [9, 10]. One major hypothesis is fetal glucocorticoid overexposure as a consequence of maternal stress [50]. 

To our knowledge, maternal predictors of birth weight variations in AGA neonates in nondiabetic, nonhypertensive pregnancies are not yet established. This study highlights that increases in maternal postload sugar levels and blood pressure, within the normal range, highly predict the outcome of NBW in AGA neonates. This is particularly apparent in the results showing that mild increases in postload glucose levels determine marked increases in NBW in the absence of maternal diabetes. These findings lend importance to further explore the effect of dietary intake of healthy mothers on NBW in larger-scale studies and provide focus for early dietary intervention measures to improve maternal-neonatal outcome. 

## Figures and Tables

**Table 1:** Maternal anthropometrics and biochemical measures.

	Mean ± standard deviation	Normal values
Maternal parameters		
Age years	30.2 ± 4.5	—
Initial BMI (Kg/m^2^)	28.3 ± 7.8	—
BMI at late gestation (Kg/m^2^)	32.5 ± 7.5	—
Systolic blood pressure (mmHg)	114 ± 10.5	90–119^1^
Diastolic blood pressure (mmHg)	64.3 ± 7.2	70–79^1^
Parity (no. of pregnancies)	3.13 ± 2.2	—
Gestational period (weeks)	38.9 ± 1.6	38–42
Maternal weight gain (Kg)		
Normal weight women: BMI > 18.5 (*n* = 14)	11.7 ± 4.2	11.3–15.8^2^
Obese: BMI > 30 (*n* = 22)	9.0 ± 5.4	6.8–11.3^2^
Fasting glucose (mmol/L)	4.8 ± 0.5	<5.1^3^
2 hr postload glucose (mmol/L)	6.3 ± 0.95	<7.8^3^
Triglycerides (mmol/L)	2.25 ± 0.78	<1.7^3^
LDL-C (mmol/L)	3.9 ± 1.4	<2.6^3^
HDL-C (mmol/L)	1.64 ± 0.39	>1.3^3^
Infant characteristics		
Neonatal birth weight (weight Kg)	3.17 ± 0.26	2.5–4^4^
Males (weight Kg)^§^ (*n* = 17)	3.2 ± 0.2^NS^	2.5–4^4^
Females (weight Kg) (*n* = 19)	3.1 ± 0.29	

^§^There are no established differences between neonate males and females at birth.

^NS^No significant differences were found between neonate males and females at birth in this study.

^1^[51], ^2^[35], ^3^[52], and ^4^[34].

**Table 2:** Comparisons of maternal parameters by one way analysis of variance (ANOVA) across neonatal birth weight tertiles.

Neonatal birth weight tertiles* (mean weight ± SD)	1st tertile (2.89 ± 0.1)	2nd tertile (3.16 ± 0.07)	3rd tertile (3.44 ± 0.19)	*P*
Maternal parameters				
2 hr postload glucose (mmol/L)	5.94 ± 0.77	5.99 ± 0.89	6.84 ± 0.97	0.029*
Fasting glucose (mmol/L)	4.77 ± 0.67	4.77 ± 0.42	4.95 ± 0.38	0.62
Initial BMI (Kg/m^2^)	27.2 ± 8.0	28.4 ± 9.1	29.3 ± 6.6	0.81
Systolic blood pressure (mmHg)	118.5 ± 11.5	114.2 ± 6.8	109.2 ± 10.9	0.09
Diastolic blood pressure (mmHg)	66.9 ± 7.1	64 ± 7.7	62 ± 6.7	0.26
BMI at late gestation (Kg/m^2^)	31.4 ± 8.3	32.1 ± 8.0	33.9 ± 6.2	0.71
Maternal weight gain (Kg)	10.1 ± 5.6	9.07 ± 4.5	10.9 ± 5.4	0.68
Triglycerides (mmol/L)	2.27 ± 0.68	2.20 ± 1.0	2.3 ± 0.57	0.96
Age (years)	30.9 ± 5.07	28.1 ± 4.1	31.6 ± 3.7	0.13

Birth weight range (2.7–3.9 Kg).

*Tertiles (weight range (Kg): 1st (2.70–3.04, *n* = 12), 2nd (3.05–3.24, *n* = 12), 3rd (3.25–3.90, *n* = 12).

Tukey's test was used for post hoc comparison of means between each pair of groups.

Results presented as mean ± standard deviation, significance *P* < 0.05.

**Table 3:** Bivariate correlation between maternal parameters and fetal birth weight.

Maternal parameter	Correlation coefficient	
	*R* value	*P* value
2 hr postload glucose	0.402*	0.015
Systolic blood	−0.40*	0.02
Diastolic blood	−0.26	0.13
Fasting glucose	0.16	0.35
BMI (first trimester)	0.03	0.89
BMI (late gestation)	0.05	0.77
Maternal weight gain	0.06	0.75
Triglycerides	0.08	0.62
Age	0.09	0.61

*Significant correlation with fetal birth weight. *P* < 0.05.

**Table 4:** Multiple regression summary for neonates appropriate for gestational age (APA).

Model		Beta	*R* ^2^	*P*
1	2-hr postload glucose	0.401	0.16	0.017

2	2-hr postload glucose	0.402	0.357	0.003*
	Systolic blood pressure	−0.447		0.004*

Dependent variable: neonatal birth weight (NBW). Independent variables: maternal age, initial BMI, weight gain, serum TG, cholesterol, LDL cholesterol, HDL cholesterol, fasting glucose and postload glucose levels, and systolic and diastolic blood pressure. *Significance set at *P* < 0.05.

## References

[1] Han JC, Lawlor DA, Kimm SY (2010). Childhood obesity. *The Lancet*.

[2] Ding EL, Hu FB (2008). Determining origins and causes of childhood obesity via mendelian randomization analysis. *PLoS Medicine*.

[3] Ludwig D (2007). Childhood obesity—the shape of things to come. *The New England Journal of Medicine*.

[4] Ludwig D, Currie J (2010). The association between pregnancy weight gain and birthweight: a within-family comparison. *The Lancet*.

[5] Krebs NF, Jacobson MS, the American Academy of Pediatrics Committee on Nutrition (2003). Prevention of pediatric overweight and obesity. *Pediatrics*.

[6] Ay L, Kruithof CJ, Bakker R (2009). Maternal anthropometrics are associated with fetal size in different periods of pregnancy and at birth. The generation R study. *International Journal of Obstetrics and Gynaecology*.

[7] Maresh M (2005). Screening for gestational diabetes mellitus. *Seminars in Fetal and Neonatal Medicine*.

[8] Sacks D (2010). Big babies in pregnancies complicated by diabetes: is it all about maternal glucose?. *Perinatology*.

[9] Churchill D, Perry IJ, Beevers DG (1997). Ambulatory blood pressure in pregnancy and fetal growth. *The Lancet*.

[10] Yadav H, Lee N (2013). Maternal factors in predicting low birth weight babies. *Medical Journal of Malaysia*.

[11] Schaefer-Graf UM, Graf K, Kulbacka I (2008). Maternal lipids as strong determinants of fetal environment and growth in pregnancies with gestational diabetes mellitus. *Diabetes Care*.

[12] Ong KK, Diderholm B, Salzano G (2008). Pregnancy insulin, glucose, and BMI contribute to birth outcomes in nondiabetic mothers. *Diabetes Care*.

[13] Farmer G, Russell G, Hamilton-Nicol DR (1988). The influence of maternal glucose metabolism on fetal growth, development and morbidity in 917 singleton pregnancies in nondiabetic women. *Diabetologia*.

[14] Bonomo M, Corica D, Mion E (2005). Evaluating the therapeutic approach in pregnancies complicated by borderline glucose intolerance: a randomized clinical trial. *Diabetic Medicine*.

[15] Jensen DM, Damm P, Sørensen B (2001). Clinical impact of mild carbohydrate intolerance in pregnancy: a study of 2904 nondiabetic Danish women with risk factors for gestational diabetes mellitus. *American Journal of Obstetrics and Gynecology*.

[16] Catalano PM, Thomas A, Huston-Presley L, Amini SB (2003). Increased fetal adiposity: a very sensitive marker of abnormal in utero development. *American Journal of Obstetrics and Gynecology*.

[17] Fraser A, Tilling K, MacDonald-Wallis C (2010). Association of maternal weight gain in pregnancy with offspring obesity and metabolic and vascular traits in childhood. *Circulation*.

[18] Upadhyay S, Biccha RP, Sherpa MT, Shrestha S, Panta PP (2011). Association between maternal body mass index and the birth weight of neonates. *Nepal Medical College Journal*.

[19] Merchant SS, Momin IA, Sewani AA, Zuberi NF (1999). Effect of prepregnancy body mass index and gestational weight gain on birth weight. *The Journal of the Pakistan Medical Association*.

[20] Asplund CA, Seehusen DA, Callahan TL, Olsen C (2008). Percentage change in antenatal body mass index as a predictor of neonatal macrosomia. *Annals of Family Medicine*.

[21] Blackburn ST, Loper DL, Eoyang T (1992). Carbohydrate, fat, and protein metabolism. *Maternal, Fetal and Neonatal Physiology: A Clinical Perspective*.

[22] Sekhavat L, Zare F, Akhavan Karbasi S (2008). Maternal serum triglyceride at midpregnancy and newborn weight in nondiabetic and normal BMI women. *Nepal Journal of Obstetrics and Gynaecology*.

[23] Saleh J, Al-Riyami H, Chaudhary T, Cianflone K (2008). Cord blood ASP is predicted by maternal lipids and correlates with fetal birth weight. *Obesity*.

[24] Di Cianni G, Miccoli R, Volpe L (2005). Maternal triglyceride levels and newborn weight in pregnant women with normal glucose tolerance. *Diabetic Medicine*.

[25] Knopp RH, Bonet B, Zhu X, Cowett RM (1988). Lipid metabolism in pregnancy. *Principles of Perinatal-Neonatal Metabolism*.

[26] Kitajima M, Oka S, Yasuhi I, Fukuda M, Rii Y, Ishimaru T (2001). Maternal serum triglyceride at 24–32 weeks’ gestation and newborn weight in nondiabetic women with positive diabetic screens. *Obstetrics and Gynecology*.

[27] Hernandez TL, Friedman JE, Van Pelt RE, Barbour LA (2011). Patterns of glycemia in normal pregnancy: should the current therapeutic targets be challenged?. *Diabetes Care*.

[28] Metzger BE, Lowe LP, Dyer AR (2008). HAPO study cooperative research group hyperglycemia and adverse pregnancy outcomes. *The New England Journal of Medicine*.

[29] Kerényi Z, Tamás G, Kivimäki M (2009). Maternal glycemia and risk of large-for-gestational-age babies in a population-based screening. *Diabetes Care*.

[30] Breschi MC, Seghieri G, Bartolomei G, Gironi A, Baldi S, Ferrannini E (1993). Relation of birthweight to maternal plasma glucose and insulin concentrations during normal pregnancy. *Diabetologia*.

[31] Jovanovic-Peterson L, Peterson CM, Reed GF (1991). Maternal postprandial glucose levels and infant birth weight: the diabetes in early pregnancy study. *American Journal of Obstetrics and Gynecology*.

[32] Mello G, Parretti E, Cioni R (2003). The 75-gram glucose load in pregnancy: relation between glucose levels and anthropometric characteristics of infants born to women with normal glucose metabolism. *Diabetes Care*.

[33] World Health Organization (2006). *Definition and Diagnosis of Diabetes Mellitus and Intermediate Hyperglycemia: Report of a WHO/IDF Consultation*.

[34] The NS, Adair LS, Gordon-Larsen P (2010). A study of the birth weight-obesity relation using a longitudinal cohort and sibling and twin pairs. *American Journal of Epidemiology*.

[35] http://www.webmd.com/baby/guide/healthy-weight-gain.

[36] Sermer M, Naylor CD, Gare DJ (1995). Impact of increasing carbohydrate intolerance on maternal-fetal outcomes in 3637 women without gestational diabetes. *American Journal of Obstetrics and Gynecology*.

[37] Sacks DA, Greenspoon JS, Abu-Fadil S, Henry HM, Wolde-Tsadik G, Yao JF (1995). Toward universal criteria for gestational diabetes: the 75-gram glucose tolerance test in pregnancy. *American Journal of Obstetrics and Gynecology*.

[38] Tallarigo L, Giampietro O, Penno G (1986). Relation of glucose tolerance to complications of pregnancy in nondiabetic women. *The New England Journal of Medicine*.

[39] Little RR, McKenzie EM, Shyken JM (1990). Lack of relationship between glucose tolerance and complications of pregnancy in nondiabetic women. *Diabetes Care*.

[40] Langhoff-Roos J, Wibell L, Gebre-Medhin M, Lindmark G (1989). Placental hormones and maternal glucose metabolism: a study of fetal growth in normal pregnancy. *British Journal of Obstetrics and Gynaecology*.

[41] Breschi MC, Seghieri G, Bartolomei G, Gironi A, Baldi S, Ferrannini E (1993). Relation of birthweight to maternal plasma glucose and insulin concentrations during normal pregnancy. *Diabetologia*.

[42] Louie JCY, Brand-Miller JC, Markovic TP, Ross GP, Moses RG (2010). Glycemic index and pregnancy: a systematic literature review. *Journal of Nutrition and Metabolism*.

[43] Pedersen J (1954). Weight and length at birth of infants of diabetic mothers. *Acta Endocrinologica*.

[44] Langer O (2000). Fetal macrosomia: etiologic factors. *Clinical Obstetrics and Gynecology*.

[45] Lager S, Powell TL (2012). Regulation of nutrient transport across the placenta. *Journal of Pregnancy*.

[46] Nolan CJ, Riley SF, Sheedy MT, Walstab JE, Bescher NA (1995). Maternal serum triglyceride, glucose tolerance, and neonatal birth weight ratio in pregnancy. *Diabetes Care*.

[47] Grundt JH, Nakling J, Eide GE, Markestad T (2012). Possible relation between maternal consumption of added sugar and sugar-sweetened beverages and birth weight–time trends in a population. *BMC Public Health*.

[48] Nucci LB, Duncan BB, Mengue SS, Branchtein L, Schmidt MI, Fleck ET (2001). Assessment of weight gain during pregnancy in general prenatal care services in Brazil. *Cadernos de Saude Publica*.

[49] Kayemba-Kay’s S, Peters C, Geary MP, Hill NR, Mathews DR, Hindmarsh PC (2013). Maternal hyperinsulinism and glycaemic status in the first trimester of pregnancy are associated with the development of pregnancy-induced hypertension and gestational diabetes. *European Journal of Endocrinology*.

[50] Khulan B, Drake AJ (2012). Glucocorticoids as mediators of developmental programming effects. *Clinical Endocrinology and Metabolism*.

[51] American Heart Association (2011). *Understanding Blood Pressure Readings*.

[52] Painter P, Cope J, Smith J, Burtis C, Ashwood E (1999). Reference information for the clinical laboratory. *Tietz Text Book of Clinical Chemistry*.

